# Detection and Characterization of Histamine-Producing Strains of *Photobacterium damselae* subsp. *damselae* Isolated from Mullets

**DOI:** 10.3390/vetsci4020031

**Published:** 2017-06-20

**Authors:** Marcello Trevisani, Rocco Mancusi, Matilde Cecchini, Claudia Costanza, Marino Prearo

**Affiliations:** 1 Dipartimento di Scienze Mediche Veterinarie, Università degli Studi di Bologna, Alma Mater Studiorum, via Tolara di Sopra 50, Ozzano Emilia 40064, Italy; mancusirocco@virgilio.it (R.M.); matilde.cecchini@unibo.it (M.C.); claudia.costanza.vet@gmail.com (C.C.); 2 S.S. Laboratorio Specialistico Ittiopatologia, Istituto Zooprofilattico Sperimentale del Piemonte, Liguria e Valle d’Aosta, Torino 10154, Italy; Marino.Prearo@izsto.it

**Keywords:** *Photobacterium damselae* subsp. *damselae*, histidine decarboxylase, cross contamination, mullets, histamine biosensor

## Abstract

*Photobacterium damselae* subsp. *damselae* (*Pdd*) is considered to be an emerging pathogen of marine fish and has also been implicated in cases of histamine food poisoning. In this study, eight strains isolated from mullets of the genera *Mugil* and *Liza* captured in the Ligurian Sea were characterized, and a method to detect histamine-producing *Pdd* from fish samples was developed. The histamine-producing potential of the strains was evaluated in culture media (TSB+) using a histamine biosensor. Subsequently, two strains were used to contaminate mackerel fillets (4 or 40 CFU/g), simulating a cross-contamination on the selling fish stalls. Sample homogenates were enriched in TSB+. The cultures were then inoculated on thiosulfate-citrate-bile salts-sucrose agar (TCBS) and the dark green colonies were cultured on Niven agar. The violet isolates were characterized using specific biochemical and PCR based tests. All *Pdd* strains were histamine producers, yielding concentration varying from 167 and 8977 µg/mL in TSB+ cultures incubated at 30 °C for 24 h. *Pdd* colonies were detected from the inoculated mackerel samples and their histidine decarboxylase gene was amplified using species-specific primer pairs designed for this study. The results indicate that mullets can be source of *Pdd* and the fish retailers needs to evaluate the risk posed by cross-contamination on the selling fish stalls.

## 1. Introduction

Histamine fish poisoning is among the most common food borne diseases related to fish consumption. Fifty-six of the 71 food borne disease outbreaks (78.9%) that have been notified in Europe in 2011 were due to histamine fish poisoning [1]. The risk is correlated with the number and the histidine decarboxylase activity of the contaminating bacteria that grow in the flesh of fishes that are rich of free histidine, such as tuna, mackerel, and bonito.

Bacteria of the genus *Photobacterium*, i.e., *P. damselae* subsp. *damselae* (*Pdd*) and *P. phosphoreum*, are strong histamine producers [2,3,4].

*Photobacterium damselae* subsp. *damselae* is considered to be an emerging pathogen of marine fish of importance in aquaculture, with a notable increase in its geographical distribution during the last several years [5].

Kanki et al. [2] demonstrated that *Pdd* inoculated on tuna can produce toxic levels of histamine even at 4 °C. These authors observed that *Pdd* displayed the highest performance in accumulating histamine in fish samples stored at refrigeration temperature in comparison with other psychrotolerant marine bacteria, namely *P. phosphoreum* and *Raoultella planticola*. They demonstrated that *Pdd* (strain JCM 8968) can produce more than 500 mg/kg histamine at 4 °C in 24 h and maintain 60% and 50% of the initial activity in tuna and dried saury for up to 12 weeks at −20 °C, respectively. The presence of *Pdd* in the fish that are stored in melting ice or at chilling temperature and even in the de-frozen and processed seafood can thus pose a significant hazard if the contamination is carried on fish species which are rich in free histidine.

The histidine decarboxylase activity of bacteria that are present in fish samples can be assessed in enrichment broth supplemented with histidine using different analytical methods, including immuno-enzymatic (ELISA) tests, chromatographic methods, or bio-sensing devices [3,6,7,8]. The detection of histamine-producing bacteria (HPB) is possible by plating the fish homogenates on differential grow media, such as the Niven agar plates [9] and the screening of the suspect colonies with PCR assays for the histidine decarboxylase encoding genes [10], but these analytical methods did not allow the isolation of HPB in a number of samples that developed high level of histamine in the enrichment broth supplemented with histidine [7]. Many studies have demonstrated that among HPB there are some species that are not able to grow on the Niven medium and consequently false negative results occur [11]. In order to isolate the halophilic *Photobacterium* spp., the use of specific culture medium is needed.

There are few studies concerning the histidine-decarboxylase encoding genes of *P. damselae* and *P. phosphoreum* [11], and the variability of phenotype expression (histidine-decarboxylase activity) [3,4]. Biosensor technology allows fast, cost effective, and specific detection of histamine in seafood spoilage [12] and histamine biosensors can be useful in assessing the microbiological quality of fish. The enzyme diaminoxidase is known to catalyse the conversion of histamine to imidazole acetaldehyde by means of a bi-enzyme system, used by various researchers [13,14,15]. The co-substrate, molecular oxygen, is reduced to hydrogen peroxide (Equation (1)). The reduction of hydrogen peroxide to water is catalysed by the enzyme peroxidase using potassium hexacyanoferrate(II) (or other redox systems) as the mediator (Equation (2)). The introduction of a mediator in the bi-enzymatic system (DAO/HRP) leads to an acceleration of the electron transfer, a decrease in the applied working potential (the reduction of the mediator is detected at the electrode), and an increase in sensitivity [15,16,17].

RCH_2_NH_2_ + O_2_ + H_2_O → (DAO) → RCHO + NH_3_ + H_2_O_2_ (1)

H_2_O_2_ + Fe(CN)_6_^−4^ → HRP → H_2_O + Fe(CN)_6_^−3^ + e^−^ (2)

The working electrode of a potentiostat acts as an oxidant and the oxidation current increases. The aims of this study were to develop a simple and rapid method to detect histamine-producing *P. damselae* subsp. *damselae* from fish samples and characterize their histidine decarboxylase activity.

## 2. Materials and Methods 

### 2.1. Characterization of Pdd Strains

Eight strains of *Pdd* that have been isolated from different samples of fish of the species *Mugil cephalus*, *Liza aurata*, *L. ramada,* and *L. saliens*, which were captured at the estuary of the river Magra in the Eastern Ligurian Sea (Bocche di Magra and Fiumaretta, Amelia municipality, La Spezia province, Italy) [18], were used in this study. The isolates were characterized with biochemical tests for their oxidase, catalase, urease activities, and the ability of fermenting glucose and galactose. In addition, the presence of specific target genes *ure*C and *16S* rRNA was assessed by PCR using the primers designed by Osorio et al. [19] (Table 1).

In order to detect the histidine decarboxylase gene of *P. damselae* subsp. *damselae* two primer pairs were used. The primers *HIS*2-F and *HIS*2-R designed by De Las Rivas et al. [10] allow the amplification of a specific 531-bp DNA fragment from gram-negative histamine-producing bacteria including *P. damselae*, strain CECT 626T (ATCC 33539) [20].

In this study, target-specific primers for *Pdd* were designed to be specific for Genbank accession AB259289.1 (HDC, Histidine decarboxylase gene of *Photobacterium damselae* (strain JCM 8968)) [2]. The designed PCR primers (*hdc*Pdd, Table 1) were checked for specificity with Primer-Blast software (NCBI, Cambridge, UK).

### 2.2. DNA Extraction and PCR Procedures

Strains were grown on Tryptone Soy Agar (TSA, Thermofisher, Milano, Italy) supplemented with NaCl (2%) at 25 °C for 48 h. Two colonies were randomly picked for each strain using a sterile toothpick and the DNA was extracted by boiling suspensions of cells in a 5% suspension of Chelex 100 (Bio-Rad Laboratories, Hercules, CA, USA) following the producer protocol.

Real-Time PCR was performed on a MiniOpticon Real-Time PCR System (Bio-Rad Laboratories) using SsoFast EvaGreen Supermix (Bio-Rad). Total reaction volume of 20 μL included: 300 nM of each forward and reverse primers, 1 µL of template DNA and the SsoFast EvaGreen Supermix (according to the manufacturer’s instructions). Each run included a non-template control (NTC). Two laboratory reference strains characterized as *Morganella morganii* and *Photobacterium damselae* subsp. *damselae* in accordance with their colony characteristics, biochemical tests (API20E and API20NE systems) and genetic characterization were used as positive controls [19,21]. Real-time amplification for each gene, including controls and samples, was performed in duplicate under identical PCR conditions. Thermal cycle was set as reported in Table 1. At the end of the PCR cycles, a melting curve was conducted between 65 °C and 95 °C with a 0.5 °C/5 s increment read and continuous fluorescence measurement. The effect of annealing temperature was analyzed using gradient PCR amplification in the range from 49 to 53 °C.

### 2.3. Assay for Histamine Production: A Biosensor Fabrication

To confirm histamine production by each HPB isolate, single colonies were suspended in Tryptone Soy Broth (Thermofisher, Milano, Italy) supplemented with NaCl (2%) and L-Histidine hydrochloride (1%) (Sigma-Aldrich, Milano, Italy) (TSB+) and incubated at 30 °C for 24 h. After the incubation, the cultures were sterilized at 121 °C for 15 min and the supernatant was used for histamine quantitative detection.

To comparatively assess histamine production ability of each strain, the microbial suspensions were diluted with TSB+ to have 40% optical density at 540 nm, corresponding to approximately 10^8^ CFU/mL. The concentration of bacteria was subsequently assessed by plating the appropriate dilutions on Tryptone Soy Agar (TSA, Thermofisher, Milano, Italy) with 2% NaCl.

Quantitative detection of histamine in the enriched broth cultures and standardized microbial suspensions was made using an amperometric enzymatic biosensor. The histamine biosensor is based on the co-immobilization diamine-oxidase (DAO, from Porcine Kidney, 0.11 U/mg) and peroxidase (HRP, from horseradish, 25 KU/mg) (Sigma-Aldrich, Milano, Italy) on the surface of a glassy carbon electrode (diameter 3 mm, BAS, West Lafayette, IN, USA). The enzymes were immobilized by chemical cross-linking with glutaraldehyde (GA, 25%) and bovine serum albumin (BSA, fraction V, purity 96–99%). All reagents were purchased from Sigma-Aldrich (Milano, Italy). Briefly, 10 mg of DAO, 6 mg of HRP and 4 mg of BSA were dissolved in 100 µL of Phosphate Buffer (PB, 0.1 M Ph = 7.4). Five µL of GA diluted in water (2.5%) were mixed with 15 µL of the DAO-HRP-BSA solution. Ten µL of the final solution was put on the cleaned surface of the working electrode and allowed to dry for 30 min at 25 °C. After the BSA enzyme solution were cross-linked with GA and a consistent gel has developed on the electrode surface, the enzymatic sensor was washed in PB and stored in the same medium at refrigeration temperature.

The response of the biosensor to the histamine concentration in the bacterial cultures was calculated as the difference between the amperometric signal in inoculated and non-inoculated medium. The values were recorded in millivolts by a potentiostat (Metrhom Autolab PGSTAT10, EcoChemie, Utrecht, The Netherlands) connected to a personal computer using the Autolab GPES 4.9 software. The calibration data was obtained with a series of standard histamine solutions (1, 2, 4, 6, 8, 10, and 20 ppm) were fitted using the software Excel (Microsoft, Redmond, WA, USA). The concentration of histamine in the enriched cultures was calculated by interpolation of the amperometric responses for the samples into the calibration plot constructed with histamine standards.

### 2.4. Detection of *Pdd* from Fish Samples

A procedure to detect *Pdd* in fish was developed and tested. With this aim mackerel samples were inoculated in duplicate with two *Pdd* strains that produced more than 1000 µg/mL of histamine in TSB+ culture incubated at 30 °C for 24 h (high HPB). The test were carried out independently with each of the two strains. Fresh mackerels were purchased from the market and samples (25 g) were inoculated with either 100 µL of *Pdd* suspensions standardized to approximately 10^3^ and 10^4^ CFU/mL (4 and 40 CFU/g) and control samples (taken from the same mackerels) were analyzed in parallel (controls).

The samples were homogenized (1:10 w/v) in TSB+ and the contaminating bacteria were enriched at 25 °C for 48 h and subsequently plated on thiosulfate-citrate-bile salts-sucrose agar (TCBS, Thermofisher, Milano, Italy). Dark green colonies with smooth edges were picked and inoculated on plates of modified Niven Agar [9]. Isolated purple colonies developed within 48 h (suspect HPB) were picked and seeded on TSA with 2% NaCl.

Isolates were analyzed for the oxidase activity (oxidase detection strips Oxoid, Thermofisher, Milano, Italy) and by Gram stain, then the Gram-oxidase positive strains were characterized for the ability to ferment glucose and galactose and the urease activity with the API 20E kit (bioMérieux Italia, Bagno di Ripoli, FI, Italy). DNA of the strains that gave positive reactions was extracted by boiling suspensions of cells in a 5% suspension of Chelex 100 (Bio-Rad Laboratories) following the producer protocol. DNA extracts were analyzed to detect the presence or absence of specific nucleotide sequence of the genes *ure*C e *16S* rRNA of *Pdd* by using the PCR protocols described (Table 1).

## 3. Results

**Figure 1:**
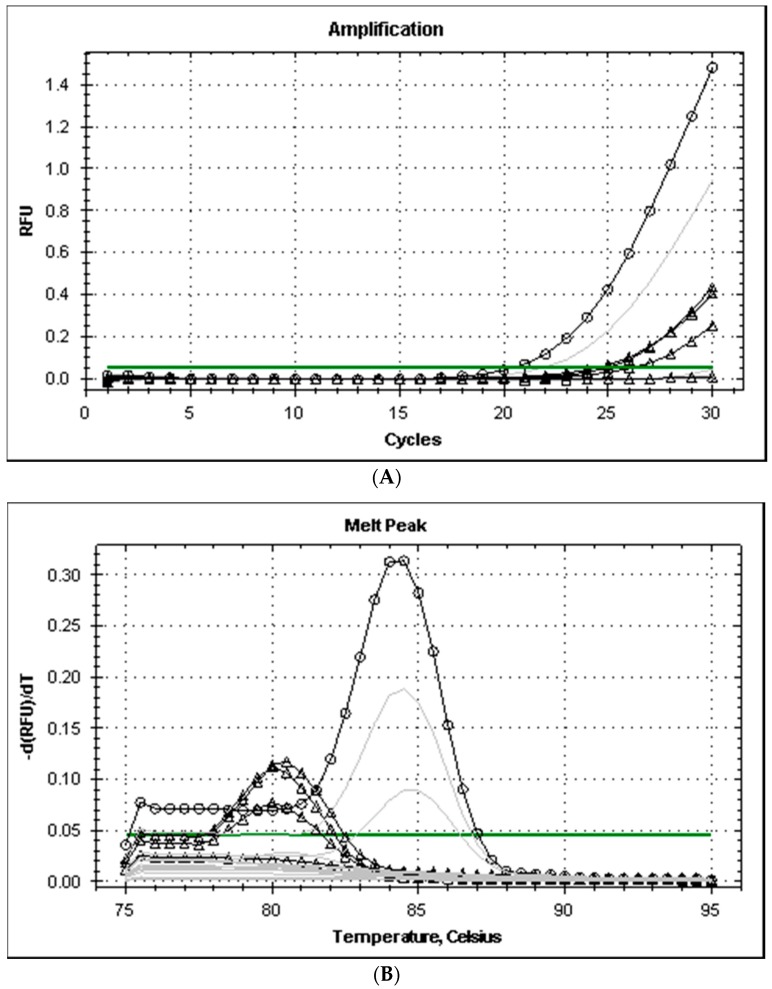
Amplification plot (**A**) and melt curves (**B**) from rt-PCR of histidine decarboxylase genes of Gram-negative bacteria (primers hdc-dp). Legend: annealing temperature for gradient PCR amplification ranged from 49 (dark lines) to 53 °C (grey lines); lines with symbols Δ and ○ correspond to *Pdd* (3 strains)and *M. morgani*, respectively.

All the strains isolated from the fish of the genera *Mugil* and *Liza* captured in the Ligurian Sea grew on TCBS producing dark green colonies with smooth edges, tested positive at the glucose, galactose, and urease tests, and produced typical amplicons after PCR, with melting temperatures (*Tm*) of 84 °C and 86 °C for the genes *ure*C and *16S* rRNA, respectively. The primers *his*1 and *his*2 produced positive PCR tests only with three of the eight strains analyzed and the level of amplification (cycle threshold, Ct values was higher than 33). The *Tm* of the amplicons of *Pdd* was 80 °C and was different from the amplicon produced by *M. morganii* (Tm 84.5 °C) (Figure 1). The level of amplification improved when the annealing temperature of PCR was reduced from 53 to 49 °C and amplicon melting temperature remained at 80 °C for *Pdd* strains.

**Figure 2:**
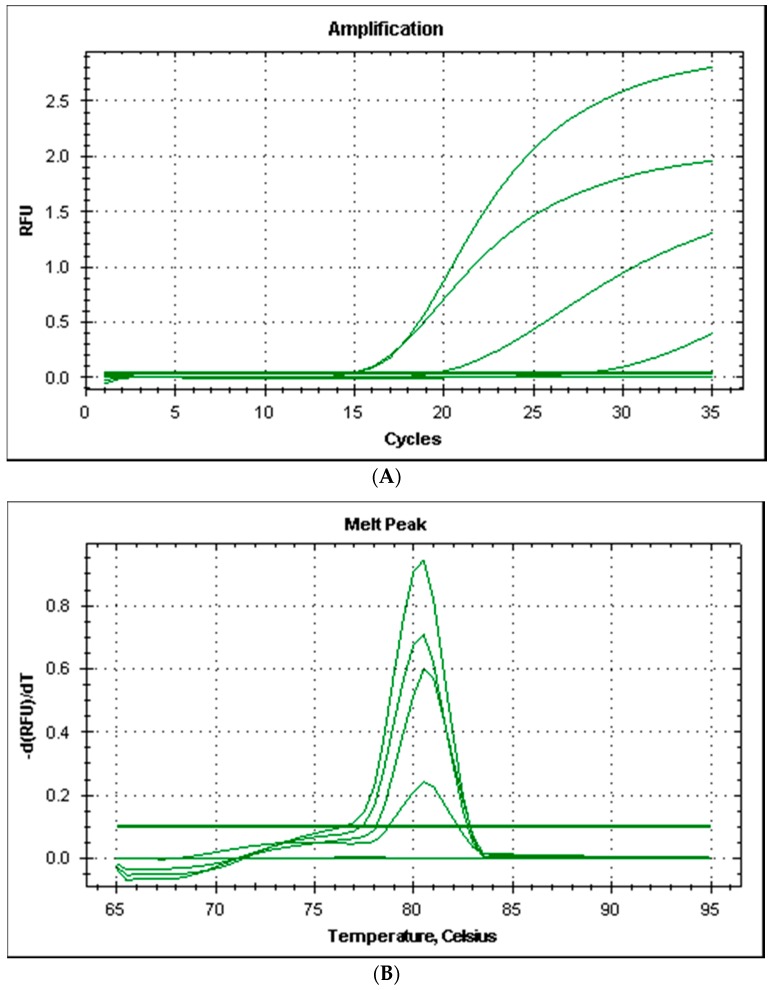
Amplification plot (**A**) and melt curves (**B**) from rt-PCR of histidine decarboxylase gene of *Photobacterium damselae* subsp. *damselae*.

All the strains produced good amplification levels by using the primers couple HDC*Pdd* designed for this study and the PCR amplicons produced had a melting temperature of 80.5 °C (Figure 2).

Molecular sequence analysis with BLAST software were used to compare the HDC*Pdd* primers sequences with the genomes of Gram-bacteria. The primers showed 100% similarity with a known nucleotide sequence of *Pdd* histidine decarboxylase gene and no similarity with other known sequences of Gram-bacteria.

All these strains gave positive results with the assay for histamine production in TSB+ at 30 °C. The concentration of histamine produced in 24 h was between 167 and 8977 µg/mL (median = 1053).

Histamine producing *Pdd* strains were isolated from all the inoculated samples and typical colonies can be easily detected and characterized using the procedure described even at the lower concentration (4 CFU/g) used in this study. Other Gram-bacteria, subsequently identified as *Proteus* spp., grew on the TCBS and also on the Niven plates, but they did not show swarming behavior. The colonies on the TCBS plates were green, but not dark as those of *Pdd*. The PCR and biochemical assays can easily discriminate the *Pdd* strains.

## 4. Discussion

Contamination of fish meat with histamine-producing bacteria that can grow at refrigeration temperature pose a risk for the marine species that have high concentration of free histidine.

The presence of psychrototrophic bacteria that are strong producers of histamine of the genus *Morganella*, *Photobacterium* e *Raoultella* was observed in fish during surveys or in the many outbreaks of intoxication [22,23,24]. Their presence has been frequently detected also in surveys carried out in the Italian fish market [7,25].

*Photobacterium damselae* subsp. *damselae* do not grow at the temperature of melting ice, but their role in accumulation of histamine in tuna and dried saury was clearly documented and was correlated to the high stability of its histidine decarboxylase also in defrozen and dried fish [2] and can persist even if its viability is reduced [21]. Recent studies have reported a notable increase in the *Pdd* geographical distribution during the last several years, especially in marine aquaculture [5,26].

Contamination can occur after the harvest, due to manipulations, and during processing. Development of histamine is favored by the spread of bacteria and HDC resulting from the loss of integrity and the post mortem decay of natural barriers (skin, gut, and gills). The risk of histamine (scombroid) intoxication is correlated with the number of HPB and their enzymatic activity of the microbial species, other than to the temperature and time [27,28].

The screening of fish samples to detect HPB and characterize their HDC activity is of utmost importance for the food business operators (FBO) and the food safety authorities that verify the effectiveness of the FBO’s controls [29,30].

Differential culture method [9], conductance method [31], and also culture independent Real-Time PCR Method [32] have been used to detect and quantify HPB. Immunological and chromatographic methods, and also biosensors, can be used to measure the histamine produced in the culture media and distinguish the phenotype of HPB strains (i.e., strong or weak histamine producers). Biosensors offer a cheaper alternative to the immunoenzymatic methods [2] or HPLC [3]. The specific detection of HPB strains is needed to characterize the risk, but many studies have reported that the detection of HPB from enriched samples that have tested positive by the assays for histamine can be hampered by the use of the Niven differential culture media, which do not allow the growth of strict halophilic species or other species which are sensitive to its low pH, producing a number of false negative results [4,7,33]. In addition, Niven medium is not selective, producing false positive isolates that must be subsequently discriminated by PCR assays or labelled DNA probes for the *hdc* gene [4,10,34].

Multiple primers sets have been reported for amplification of the histidine decarboxylase gene from Gram-negative bacteria [10,33], but this study has revealed that the degenerate primers designed by De Las Rivas et al. [10] to amplify several similar genetic sequences of the Gram-*hdc* gene do not give a good performance with the *Pdd* strains analyzed in this study. A PCR amplification was obtained only by lowering annealing temperature, thus allowing primers with many mismatches with templates to provide amplification [35].

In order to detect *Pdd* from fish samples that have tested strongly positive by the assays for histamine, the use of a selective and differential medium like TCBS in combination with the Niven medium for the selective detection of HPB allowed improvement of the performance of the detection method and the presence of *Pdd* can be confirmed by a PCR assay targeted toward the species-specific sequence of *hdc* gene.

An excellent correlation between the histamine amount values obtained with biosensors and the histamine amount values obtained by ELISA (enzyme-linked immunosorbent assay ) and HPLC has been established [14,36,37]. Therefore, its use for screening purpose is suitable to analyze the sample enrichments and detect those contaminated with strong HPB active at low or ambient temperatures. Nevertheless, comparative analysis of HPB are usually carried out at 30 °C for 24 h and the *Pdd* isolates can be categorized as high histamine (>1000 ppm) producers (three strains), intermediate (501 to 999 ppm) histamine producers (four strains), and low histamine (126 to 500 ppm) producer (one strain) [3].

The widespread distribution of *P. damselae* subsp. *damselae* was reported in farmed fish in Spain (*Pagrus auriga*) [38] and South-eastern Black Sea (*Dicentrarchus labrax*) [5] where it has been associated with high mortalities. Sea bream and sea bass or mullet are not fish species implicated in histamine poisoning cases, but these farmed fishes are commonly exposed on the selling fish stalls close to tuna and mackerel fillets and manipulated, and even the ice used in the stalls has been reported to be contaminated with HPB [39].

## 5. Conclusions

Fish retailers and other operators should review the risk management practices employed throughout the fish processing chain in relation to controlling histamine formation in at-risk fish species and consider that other fish species, such as mullets of the genera Mugil and Liza, can be considered potential carriers of Pdd. Therefore, they must ensure good hygienic practices to prevent cross contamination.

## Figures and Tables

**Table 1:** Primers and PCR protocols used for the identification of *P. damselae* subsp. *damselae* and its histidine decarboxylase gene.

Gene/Primers	Nucleotide Sequences	Thermal Cycling Conditions	Reference
*ureC* Ure-5’ Ure-3’	5’-TCCGGAATAGGTAAAGCGGG-3’ 5’-CTTGAATATCCATCTCATCTGC-3’	95 °C 4 min; 30× (95 °C 60 s; 60 °C 60 s; 72 °C 40 s); 72 °C 5 min	Osorio et al., 2000
*16S* rRNA Car1 Car2	5’-GCTTGAAGAGATTCGAGT-3’ 5’-CACCTCGCGGTCTTGCTG-3’		
*hdc* dp ^a^ HIS2-F HIS2-R	5’-AAYTSNTTYGAYTTYGARAARGARGT-3’ 5’-TANGGNSANCCDATCATYTTRTGNCC-3’	95 °C 3 min; 35× (95 °C 15 s; 53 °C * 30 s; 72 °C 40 s); 72 °C 5 min	De Las Rivas et al., 2005
*hdc*Pdd ^b^ *hdc*Pdd-F *hdc*Pdd-R	5’-GGATTAGCGCATGGATTGGT-3’ 5’-AACGCCTAAGAAACCCCACA-3’	95 °C 3 min; 30× (95 °C 10 s; 60 °C 45 s)	(Genebank accession number JCM 8968)

Notes: ^a^ dp, degenerate primers targeting multiple sequences of the Gram-histidine decarboxylase (*hdc*) genes; ^b^ primers targeting specific sequence of *Photobacterium damselae* subsp. *damselae hdc* gene. * gradient in the range from 49 to 53 °C.
